# Application of a Chairside Anaerobic Culture Test for Endodontic Treatment

**DOI:** 10.1155/2010/942130

**Published:** 2010-12-30

**Authors:** Masahiro Yoneda, Seidai Kita, Nao Suzuki, Sonia M. Macedo, Kosaku Iha, Takao Hirofuji

**Affiliations:** ^1^Section of General Dentistry, Department of General Dentistry, Fukuoka Dental College, 2-15-1 Tamura, Sawara-Ku, Fukuoka 814-0193, Japan; ^2^Fukuoka Dental College, Medical and Dental Hospital, Fukuoka 814-0193, Japan; ^3^Uma Clínica Dental S.M.M., São Paulo, 04534-003, Brazil

## Abstract

Periapical lesions are caused by bacterial infections. The fundamental objective of endodontic treatment is to eliminate bacteria present in the root canal system because they play an important role in the development and maintenance of periapical lesions. Therefore, confirming the absence of bacteria before filling root canals is important. Anaerobic culture tests have been used in many endodontic cases, and they have brought about good treatment outcomes. These tests, however, require specific apparatuses and bacteriological techniques. Here, we report a chairside anaerobic culture test that does not require any specialized apparatuses or techniques. We also report two endodontic cases in which this simple test was used. Both patients were diagnosed with chronic purulent periapical lesions. After confirming the absence of bacteria in the root canals, they were filled with gutta-percha points. At followup, the radiolucencies showed recovery, although longterm observation is under way. From these results, the authors conclude that this simple chairside anaerobic culture test is effective for evaluating periapical lesion treatment procedures.

## 1. Introduction

Periapical lesions, which manifest both chronic and acute symptoms, are caused by bacterial infections [1, 2]. Several kinds of microorganisms are related to the initiation and progression of periapical lesions, and some of them are correlated with clinical symptoms [3, 4]. Gomes et al. [3] showed the relationship between pain and the presence of specific bacteria, such as *Prevotella and Peptostreptococcus* spp., in root canals. Sundqvist [5] reported that a special and selective environment occurs in the root canal that is attributable to the cooperative and antagonistic relationships between bacteria. 

The fundamental objective of endodontic treatment is to eliminate bacteria present in the root canal system because they play an important role in the development and maintenance of periapical lesions [6, 7]. The high percentages of failure after endodontic treatment of teeth with periapical lesions have been related to circumstances of microbial origin [8]. Refractory cases and postoperative pain (interappointment flare-ups) are often related to an ongoing overgrowth of anaerobic bacteria in the periapical area [8–10]. To prevent refractory periapical lesions, confirming the absence of endodontic bacteria before filling the root canal is important [11]. 

An anaerobic culture test is used to determine the quantity of bacteria present [12]. Several methods can be used to evaluate conditions in the periapical area, such as a bacterial culture test, a smear test, or a cell culture test. With the development of anaerobic culture techniques, an anaerobic culture test is usually applied. Sjögren et al. [13] reported that complete periapical healing occurred in 94% of the cases that yielded a negative culture. Where the samples were positive prior to root filling, the success rate of treatment was just 68%. The anaerobic culture test is used often in our clinic [14, 15]. We have also tried a bacterial test using a peptidase-detecting kit [16]. These approaches are very effective and allow for the treatment of complicated cases without recurrence for an extended period [14]. However, the anaerobic culture test requires laboratory apparatuses, such as transfer medium, pipettes, and bacteria spreaders, along with adequate bacteriological techniques. The peptidase-detecting kit is easier to handle, but it also requires a substrate kit and some apparatuses to measure the enzyme activity [16].

In this paper, a chairside anaerobic culture test not requiring any specific apparatuses or specialized technique is described. The two endodontic cases treated with this simple method are also reported.

## 2. Chairside Anaerobic Culture Test

**Figure 1:**
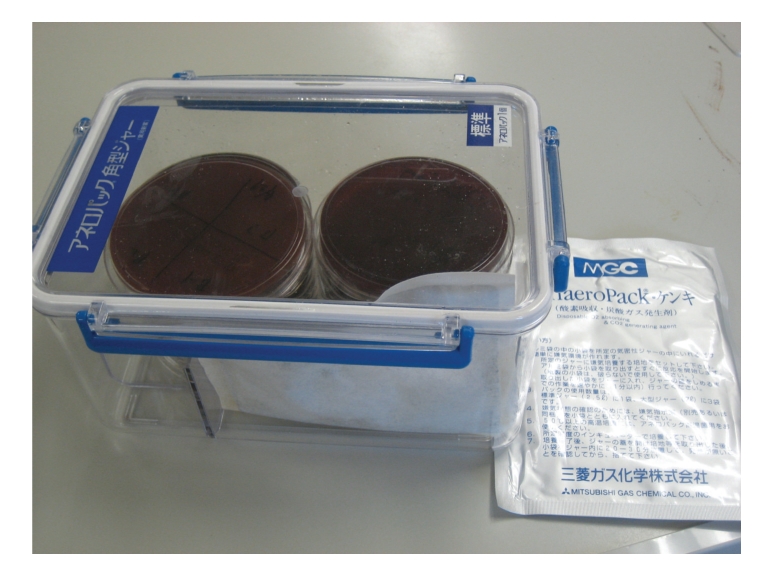
Anaerobic jar (left) and anaerobic gas-producing pouch (right). An opened pouch and blood agar plate are inside the jar.

Cavity preparation and bacterial sampling were performed by the modified method of Gomes et al. [3]. At the first visit, the cavity was prepared with an air turbine with water coolant. Then, a rubber dam was applied, and the tooth surface and rubber dam were disinfected with iodine tincture and 70% alcohol [17]. Then, the root canal chamber was opened using a low-speed engine without water coolant. A sterile paper point (#25) was introduced into the full length of the canal (as determined by a preoperative radiograph) and kept in position for 30 seconds. The paper point then was removed, and nine parallel lines were slowly drawn on the surface of a prereduced Modified-CDC anaerobe 5% Sheep Blood agar (Nippon Becton Dickinson Co. Ltd., Tokyo, Japan) plate with a chip of the paper point. The plate and an anaerobic gas-producing pouch (AnaeroPack Kenki A-03, Mitsubishi Gas Chemical Company, Inc., Tokyo, Japan) were immediately set in an anaerobic jar (Figure 1). The jar was then incubated at 37°C for 3 days. The numbers and thickness of lines made by bacterial colonies were checked at each treatment procedure. 

Root canal treatment was performed according to accepted procedures accompanied by irrigation with sodium hypochlorite. After the root canals were dressed with iodine tincture or calcium hydroxide paste (Calcipex, Nippon Shika Yakuhin Co. Ltd., Yamaguchi, Japan), the cavity was double sealed with temporary sealer (Temporary Stopping, GC Corporation, Tokyo, Japan) and glass ionomer cement. 

At subsequent dental visits, the glass ionomer cement was removed with water coolant, and the temporary sealer was removed without water coolant under a rubber dam. If Calcipex was used as a root canal dressing at the prior visit, it was carefully removed with files. The files were sometimes washed with a small amount of sterile saline to remove the calcium hydroxide paste on the instruments. If no bacterial lines or colonies were detected on the plates, then the decision was made to proceed to root canal filling.

## 3. Endodontic Cases Treated with the Chairside Anaerobic Culture Test

**Case 1**

**Figure 2:**
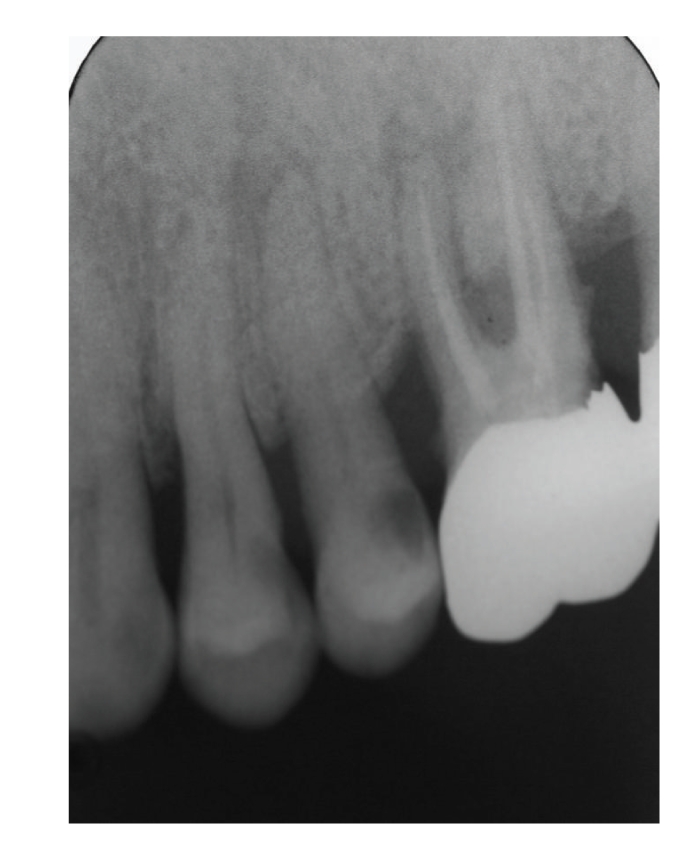
Case 1: Radiography at the first visit showing radiolucency at the apex area of tooth 46.

**Figure 3:**
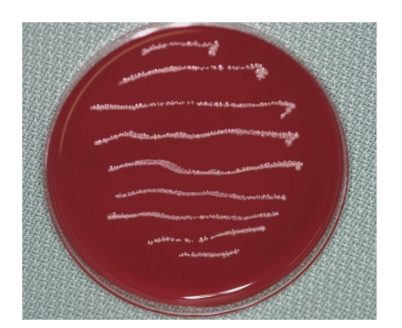
Case 1: Result of a chairside anaerobic culture test at the first visit showing thick bacterial lines.

**Figure 4:**
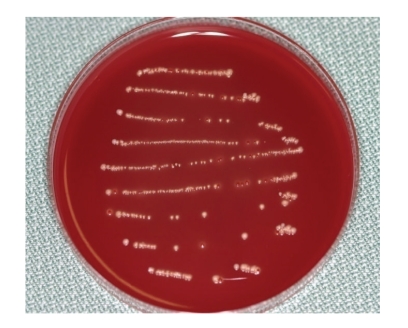
Case 1: Result of a chairside anaerobic culture test at the second visit showing thin and dotted bacterial lines.

**Figure 5:**
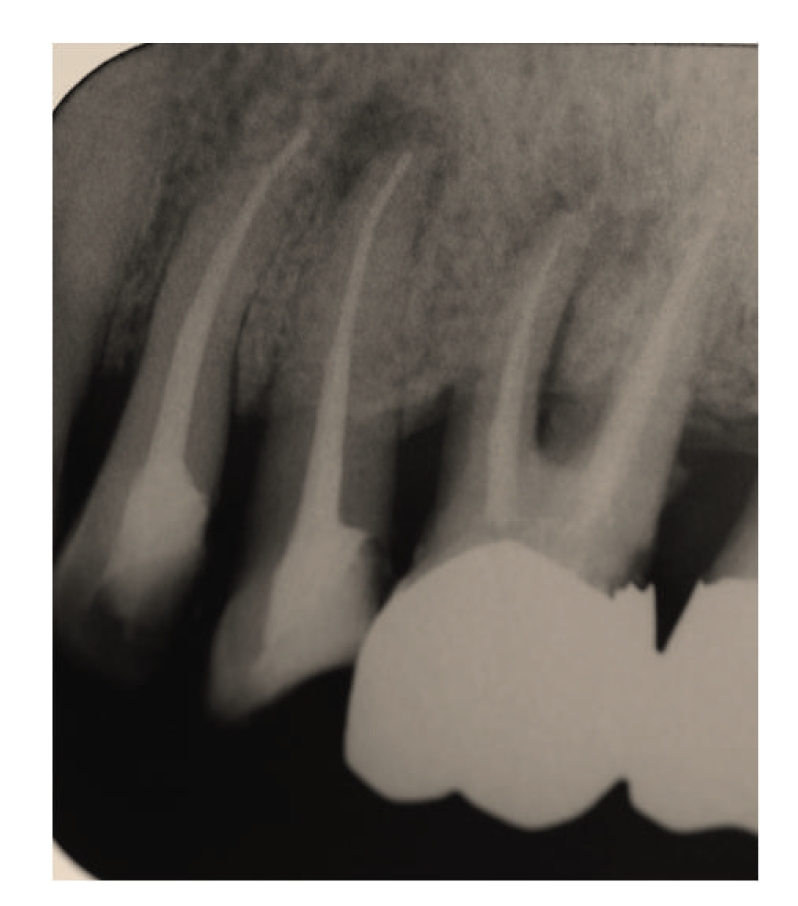
Case 1: Radiography of the root canal filling showing remaining bone loss at the root apex.

**Figure 6:**
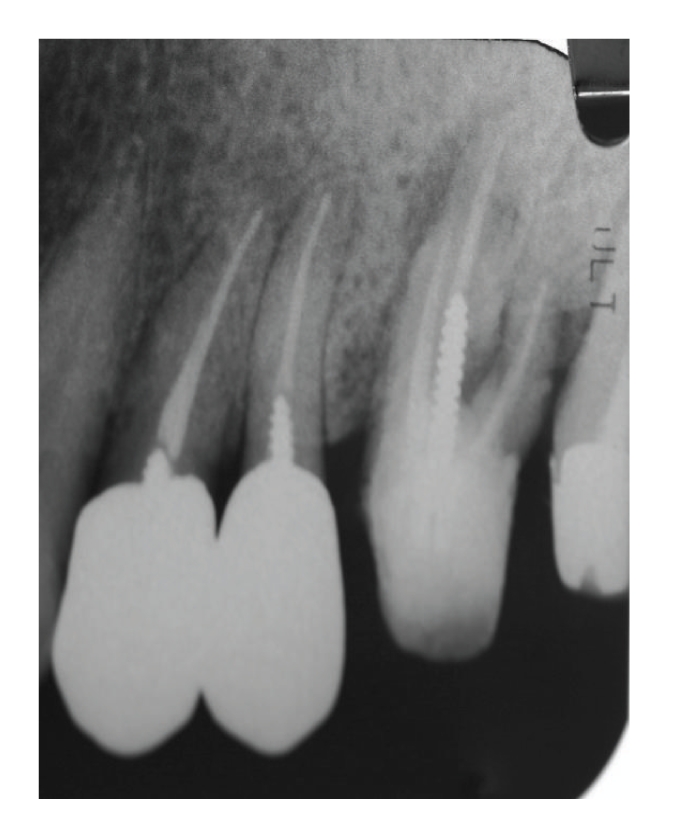
Case 1: Radiography 2 months after the initial visit showing that the radiolucency at the apex had decreased.

A 60-year-old man attended our hospital with a chief complaint of occlusal pain in the upper left molar area. Deep caries were detected in tooth 25, and radiolucency was detected in the periapical lesion (Figure 2). The patient also felt tenderness to percussion in the tooth; he was diagnosed as having chronic purulent periapical periodontitis. Minimal pus was exuded from the root canal, but the chairside anaerobic culture test revealed large amounts of bacteria in the root canal; solid lines of bacterial colonies were detected from the top to the bottom of the culture plate (Figure 3). After irrigation with sodium hypochlorite, iodine tincture was applied as the root canal dressing. At the second visit (7th day), the pus discharge had ceased, but tenderness to percussion continued. The anaerobic culture test showed some solid lines followed by dotted lines, indicating that the bacterial load was decreasing compared to the first visit (Figure 4). The root canal was enlarged to its final size, dressed with Calcipex, and double sealed. At the third visit (15th day), tenderness to percussion had disappeared, and no bacteria were detected (data not shown). The decision was made to proceed with root canal filling at the next visit. At the fourth visit (22nd day), the root canal was filled with gutta-percha points and root canal sealer (Canals, Showa Yakuhin Kako Co., Ltd., Tokyo, Japan) with a lateral condensation method (Figure 5). At this stage, clear radiolucency was still observed in the periapical area. Two months after the initial visit, the radiolucency was diminishing (Figure 6). The treatment procedure is shown in Table 1.

**Case 2**

**Figure 7:**
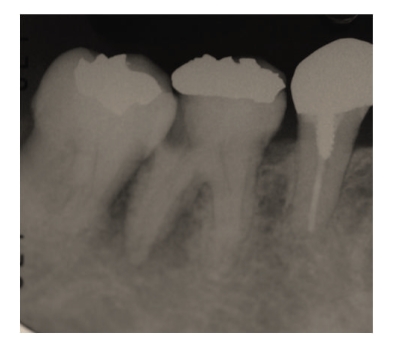
Case 2: Radiography at the first visit showing radiolucency at the apex area of tooth 46.

A 40-year-old woman visited our hospital with the chief complaint of occlusal pain in the lower right molar area. Deep caries were present in tooth 46, and a large radiolucency was observed in the periapical lesion (Figure 7). The tooth was diagnosed with a chronic purulent periapical periodontitis. Large amounts of pus possessing a rotten smell were detected when the root chamber was opened. The anaerobic culture test showed thick line-shaped colonies, indicating that the root canals were infected with large quantities of bacteria (data not shown). The root canals were irrigated with sodium hypochlorite. After dressing with iodine tincture, the canal chamber was double sealed. At the second visit, both tenderness to percussion and the quantity of bacteria had decreased (data not shown).

**Figure 8:**
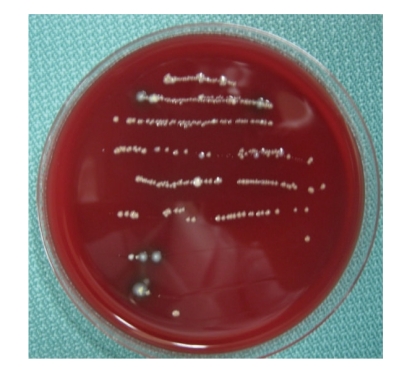
Case 2: Result of a chairside anaerobic culture test at the third visit showing several kinds of bacterial colonies including black-pigmented bacteria.

**Figure 9:**
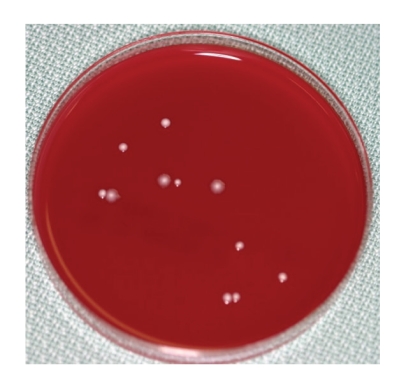
Case 2: Result of a chairside anaerobic culture test at the fourth visit showing scattered bacterial colonies.

**Figure 10:**
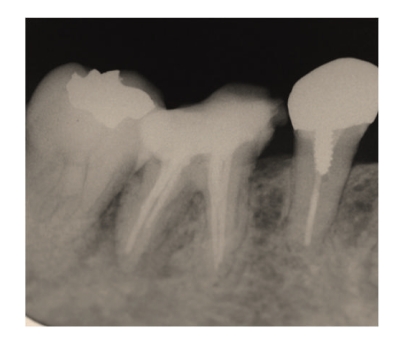
Case 2: Radiography at the root canal filling showing remaining bone loss at the root apex.

**Figure 11:**
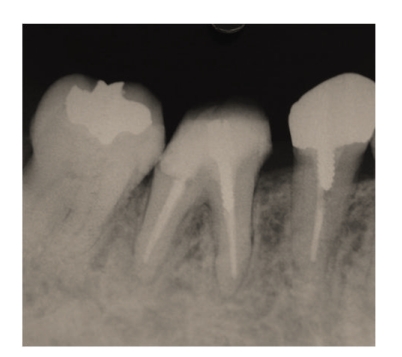
Radiography 10 weeks after the initial visit showing that the radiolucency at the apex had decreased.

At the third visit (12th day), pain had completely disappeared, but many different types of colonies remained on the culture plate (Figure 8), including black-pigmented bacteria (BPB), which indicated a mixed infection of the root canal. Irrigation with sodium hypochlorite and Calcipex dressing was continued. Pus discharge was not observed at the fourth visit (19th day), and the quantity of bacteria had decreased at this time (Figure 9). When the absence of endodontic bacteria was confirmed at the fifth visit (26th day), the root canal was filled with gutta-percha points as described above (Figure 10). Radiolucency was still observed at the apex of the distal root (Figure 10). The radiolucency was improving 10 weeks after the initial visit (Figure 11). The treatment procedure is shown in Table 2.

## 4. Discussion

The role of microorganisms in the initiation and progression of endodontic lesions has been extensively investigated in many studies [1, 2], and the importance of eliminating bacteria from root canals is beyond question. Previously, an aerobic culture system was used to confirm the absence of bacteria in root canals. However, studies have reported that 60–70% of the bacterial isolates are found to be either strict anaerobes or microaerophiles [3, 8], and an aerobic culturing technique does not sufficiently reflect the microbiological status of the canal system when used alone [4]. Therefore, the use of an anaerobic culture test with prereduced medium has been recommended [18], and good treatment outcomes have been achieved using this approach [3]. Also, reports have stated that bacterial composition and clinical symptoms are closely correlated [3, 4]. The conventional anaerobic culture method, however, requires bacteriological apparatuses and techniques. Utilizing this method in all endodontic cases may be difficult. Therefore, in addition to the conventional method, chairside anaerobic culture analysis is being performed. Several kinds of methods and apparatuses are used for this test. The procedure reported here requires no special apparatuses or bacteriological techniques, and even a trainee dentist (S. K.) could conduct this test.

This simple technique was applied in two cases of chronic purulent periapical periodontitis, and good treatment results were obtained so far. The first patient exhibited pus discharge and tenderness to percussion at the first visit, and a large quantity of bacteria was detected by the chairside anaerobic culture test. Enlargement of the root canal was not completed at the first visit because too much instrumentation may lead to flare-ups [10]. Iodine tincture was applied as a root canal dressing because the root canal was not completely enlarged at this stage and calcium hydroxide paste would not reach the root apex [19]. At the third visit, the clinical symptoms had disappeared, but root canal filling was not performed because several days were needed to obtain the results of the anaerobic culture test. After root canal filling, the prognosis seemed good by radiographic examination, and longterm follow-up is under way.

In root canal treatments without an anaerobic culture test, clinical symptoms, such as pain, exudates, and wetness of root canals, are key factors used to guide decisions about the timing of root canal filling. Therefore, if clinical symptoms have improved, the root will be filled at this point. With the anaerobic culture system, however, more dental visits are required to ensure the absence of endodontic bacteria, which may be one of the disadvantages of this system. The prognosis after root canal filling seemed good by radiographic examination, although ascertaining that the use of the anaerobic culture test brings about more rapid bone formation is impossible. The main advantage of the anaerobic culture test is considered to be a prolonged good prognosis without recurrence [13, 14].

The second patient exhibited clinical symptoms similar to those of the first patient, but more treatment time and more dental visits were required. Pus discharge continued until the third visit, and the culture test was positive until the fourth visit. The differences between the two cases may be attributable to several factors. The first case involved a single-rooted tooth and the second a multirooted tooth. The radiolucency of the second case at the first visit was larger than that of the first case, and more bacteria and tissue damage may have been present before treatment. In the second case, various kinds of colonies including black ones were observed, indicating a mixed anaerobic infection. 

The importance of anaerobic polymicrobial infection is well known [12], and the effect of BPB infection in endodontic lesion formation has been extensively reported [20–23]. Periodontal pathogenic bacteria are also known to be associated with periapical lesions, and mixed infections associated with *Porphyromonas gingivalis* have been reported to influence the progress of endodontic lesions [24]. We have examined the effect of mixed infections of *P. gingivalis* and *Tannerella forsythia* in a mouse abscess model and revealed the importance of the protease activity of *P. gingivalis* [25–27]. We applied the peptidase-detection kit to detect bacteria in root canals and obtained a good treatment outcome [16]. Another periodontal pathogen, *Fusobacterium nucleatum*, is also known to be associated with the development of severe interappointment endodontic flare-ups [28]. *P. gingivalis* is believed to enhance biofilm formation by* F. nucleatum* by releasing autoinducer-2 and other diffusible signaling molecules [29]. The chairside anaerobic culture test can detect BPB, although identifying the genera or species is impossible. 

By the simple method used in this study, anaerobic bacteria were effectively detected from root canals. Dentists can obtain useful information with this method. If the lines made by bacterial colonies are solid and thick, it means that the bacterial load is large. If the lines on the plate are narrow or dotted, bacteria may be decreasing. If only some scattered colonies are detected, it means that a bacteria-free condition is likely to be achieved soon. 

However, false-positive and false-negative results are possible. False-positive results may be caused by inappropriate handling, saliva leakage between the rubber dam and tooth during endodontic treatment, or microleakage from temporary fillings between dental visits [30]. We took great care with disinfection during the bacterial sampling and with temporary filling after each treatment, so the possibility of a false-positive result was considered minimal. We, however, cannot completely exclude the possibility of false-negative results. Calcium hydroxide paste was used as a root canal dressing, and removal of the paste was necessary before bacterial sampling. The number of bacteria may be decreased by irrigation, although minimal saline was used to clean the files. The residual calcium hydroxide may have affected the viability of bacteria on the paper point, although short-term exposure may be less inhibitory than longterm exposure [31]. Some bacterial species that do not grow directly on agar plates may have been missed because no liquid culture medium was used in this method. However, the purpose of this method is to improve the prognosis of treatment of ordinary endodontic cases, and the most important point in this system is to confirm the decrease of bacterial load at each dental visit. In more complicated cases, a conventional strict anaerobic culture test with both solid and liquid media should be applied. Moreover, to conduct a bacterial sampling without removing the calcium hydroxide paste from root canals, nonpaste dressing, such as iodine tincture, may be applied at the later stages when root canal filling is being considered [14].

The simple method described here appears to be useful for straightforward cases. However, chronic or refractory endodontic cases may be infected with certain types of bacteria, such as *Enterococcus faecalis*, or yeasts [32, 33]. Stricter bacterial identification may be necessary in the treatment of more complicated cases. In addition, some kinds of bacteria may not be culturable, even under strict anaerobic conditions [34], so other techniques such as polymerase chain reaction (PCR), checkerboard DNA-DNA hybridization, or multiplex PCR may be necessary to identify the causative bacteria in severe cases [35–37]. *E. faecalis* is known to be more frequently detected by PCR than by culture methods [38], so if periapical lesions do not respond well to endodontic treatment, PCR analysis of *E. faecalis *may be necessary.

Our findings indicate that even after the disappearance of clinical symptoms, bacteria still reside in root canals, indicating the mechanical enlargement and chemical irrigation of main canals could not easily eliminate bacteria in the accessory canals or inaccessible area. Nair et al. [39] reported that 14 of the 16 endodontically treated teeth had residual intracanal infection after instrumentation, antimicrobial irrigation, and obturation. The microbes were located in inaccessible recesses and diverticula of instrumented main canals, the intercanal isthmus, and accessory canals, mostly as biofilms. From these results, it seems dangerous to perform root canal filling in a single visit. These findings demonstrate the importance of stringent application of all nonantibiotic chemomechanical measures to treat teeth with infected and necrotic root canals to disrupt biofilms and reduce the intraradicular microbial load to the lowest possible level for a highly favorable longterm prognosis of the root canal treatment. Silveira et al. [40] also recommended multivisit treatment over single-visit treatment to eliminate the intracanal bacteria. 

Apical periodontitis is characterized by bone resorption in the periapical area. Many host defense mechanisms, including cytokine reactions, occur at the site of bacterial infection [41]. We have also examined the healing mechanisms of periapical lesions with an animal model [42]. The prognosis after endodontic treatment depends on many factors such as bacteria, cytokines, or other immune reactions [43]. Therefore, a good treatment outcome may not be solely associated with bacteria-free root canals, but the importance of bacterial elimination from the infected site should not be ignored. The chairside anaerobic culture test may contribute to a longterm good prognosis. 

Teeth that have received root canal treatment are known to lose their moisture and stiffness [44, 45], so the prognosis of endodontic treatment is also associated with restoration [46], and our former good results may be associated with proper prosthodontic treatment [14, 16]. Following up on cases in which the chairside anaerobic culture test was used and taking good care of restorations are important.

The chairside anaerobic culture test also has an educational effect for inexperienced dentists, such as trainees. We are educating trainee dentists and dental school students about the importance of correct dental treatment from the beginning of their dental careers [47]. The results of the chairside anaerobic culture test can help them understand that root canals with apical periodontitis are seriously contaminated, and they should try to eliminate bacteria under the rubber dam [48] and make good restorations. 

Many different kinds of techniques can be used to identify endodontic bacteria. Dentists should choose the best one depending on the patient's symptoms and the purpose of the examination [49]. Our findings suggest that the chairside anaerobic culture test is effective for routine endodontic cases encountered on a daily basis.

## Figures and Tables

**Table 1:** Clinical symptoms and root canal treatment.

Times of visit for dental treatment (days after initial visit)	Pus discharge from root canals	Tenderness to percussion	Colony form on agar plate	Root canal dressing
1 (0)	+	+	Thick lines	Iodine tincture
2 (7)	−	+	Dotted lines	Ca(OH)_2_
3 (15)	−	−	No colonies	Ca(OH)_2_
4 (22)	−	−	(Not done)	(Root canal filling)

**Table 2:** Clinical symptoms and root canal treatment.

Times of visit for dental treatment (days after initial visit)	Pus discharge from root canals	Tenderness to percussion	Colony form on agar plate	Root canal dressing
1 (0)	++	+	Thick lines	Iodine tincture
2 (5)	+	+	Dotted lines	Ca(OH)_2_
3 (12)	+	−	Dotted lines	Ca(OH)_2_
4 (19)	−	−	Scattered colonies	Ca(OH)_2_
5 (26)	−	−	No colonies	Ca(OH)_2_
6 (30)	−	−	(Not done)	(Root canal filling)
